# Advances in the Application of AI Robots in Critical Care: Scoping Review

**DOI:** 10.2196/54095

**Published:** 2024-05-27

**Authors:** Yun Li, Min Wang, Lu Wang, Yuan Cao, Yuyan Liu, Yan Zhao, Rui Yuan, Mengmeng Yang, Siqian Lu, Zhichao Sun, Feihu Zhou, Zhirong Qian, Hongjun Kang

**Affiliations:** 1 Medical School of Chinese PLA Beijing China; 2 The First Medical Centre Chinese PLA General Hospital Beijing China; 3 The Second Hospital Hebei Medical University Hebei China; 4 Beidou Academic & Research Center Beidou Life Science Guangzhou China; 5 Department of Radiation Oncology Fujian Medical University Union Hospital Fujian China; 6 The Seventh Affiliated Hospital Sun Yat-sen University Shenzhen China

**Keywords:** critical care medicine, artificial intelligence, AI, robotics, intensive care unit, ICU

## Abstract

**Background:**

In recent epochs, the field of critical medicine has experienced significant advancements due to the integration of artificial intelligence (AI). Specifically, AI robots have evolved from theoretical concepts to being actively implemented in clinical trials and applications. The intensive care unit (ICU), known for its reliance on a vast amount of medical information, presents a promising avenue for the deployment of robotic AI, anticipated to bring substantial improvements to patient care.

**Objective:**

This review aims to comprehensively summarize the current state of AI robots in the field of critical care by searching for previous studies, developments, and applications of AI robots related to ICU wards. In addition, it seeks to address the ethical challenges arising from their use, including concerns related to safety, patient privacy, responsibility delineation, and cost-benefit analysis.

**Methods:**

Following the scoping review framework proposed by Arksey and O’Malley and the PRISMA (Preferred Reporting Items for Systematic Reviews and Meta-Analyses) guidelines, we conducted a scoping review to delineate the breadth of research in this field of AI robots in ICU and reported the findings. The literature search was carried out on May 1, 2023, across 3 databases: PubMed, Embase, and the IEEE Xplore Digital Library. Eligible publications were initially screened based on their titles and abstracts. Publications that passed the preliminary screening underwent a comprehensive review. Various research characteristics were extracted, summarized, and analyzed from the final publications.

**Results:**

Of the 5908 publications screened, 77 (1.3%) underwent a full review. These studies collectively spanned 21 ICU robotics projects, encompassing their system development and testing, clinical trials, and approval processes. Upon an expert-reviewed classification framework, these were categorized into 5 main types: therapeutic assistance robots, nursing assistance robots, rehabilitation assistance robots, telepresence robots, and logistics and disinfection robots. Most of these are already widely deployed and commercialized in ICUs, although a select few remain under testing. All robotic systems and tools are engineered to deliver more personalized, convenient, and intelligent medical services to patients in the ICU, concurrently aiming to reduce the substantial workload on ICU medical staff and promote therapeutic and care procedures. This review further explored the prevailing challenges, particularly focusing on ethical and safety concerns, proposing viable solutions or methodologies, and illustrating the prospective capabilities and potential of AI-driven robotic technologies in the ICU environment. Ultimately, we foresee a pivotal role for robots in a future scenario of a fully automated continuum from admission to discharge within the ICU.

**Conclusions:**

This review highlights the potential of AI robots to transform ICU care by improving patient treatment, support, and rehabilitation processes. However, it also recognizes the ethical complexities and operational challenges that come with their implementation, offering possible solutions for future development and optimization.

## Introduction

### Background

Artificial intelligence (AI) and robotics are 2 distinct yet interconnected concepts ubiquitous in contemporary media and digital platforms. The term *artificial intelligence* was first introduced as a Medical Subject Heading in the US National Library of Medicine’s PubMed database in 1986, defined as “Theory and development of computer systems which perform tasks that normally require human intelligence” [1]. The hallmarks of AI encompass autonomous thinking, learning, recognition, reasoning, judgment, and inference. Medicine has long been considered a promising application field for AI, where it can augment clinical diagnostics and decision-making capacities [2]. Robotics, as defined by the Medical Subject Headings of the US National Library of Medicine, pertains to “the application of electronic, computerized control systems to mechanical devices designed to perform human functions” [3]. Presently, a standardized definition for “AI robots” remains elusive. However, they can be perceived as “physical devices inheriting electronic, computerized, and mechanical control systems, capable of perception, reasoning, learning, decision-making, and task execution without direct human control, able to mimic and execute various tasks of human intelligence” [4]. The crux of this review is the discussion of AI robot applications within the intensive care unit (ICU). While AI technology encompasses machine learning, deep learning, predictive modeling, and natural language processing, this review did not delve into these distinct technologies. Rather, it concentrated on the practical products and application cases where these AI technologies are integrated into robotic systems.

The past few decades have witnessed an exponential proliferation of research into AI robots, particularly in the health care domain. However, most of these developments have remained confined to the stages of product development and testing, with few achieving large-scale clinical implementation. The year 2020 sparked an unforeseen public health incident that catalyzed intense interest in AI robots. The outbreak of COVID-19 expedited the transformative revolution of “Healthcare + AI + Robotics” technologies and their applications [5,6]. The deployment of AI robots significantly curtailed the infection risk for medical personnel in contagion hot spots, and AI robots stood on the front lines in the battle against COVID-19 transmission [7]. Serving as a technology that improves performance, precision, and time efficiency as well as reducing costs, AI robots have promoted the upgrade and development of modern industry and are being rapidly adopted by many industries [8,9]. The applicability of robots in society is already evident and growing significantly [10].

Despite the rapid advancements in this field across military, security, transportation, and manufacturing sectors, our focus remains on intensive care scenarios such as COVID-19. ICUs are specialized settings designed to provide systematic, high-quality medical care and life-saving treatment to patients with single- or multiorgan dysfunction, life-threatening illnesses, or potential high-risk factors [11]. As scientific and technological advancements accelerate, the contradiction between the high demand for quality care for patients who are critically ill and the chronic shortage of medical resources becomes increasingly pronounced [12]. In 1995, Hanson and Marshall [13] postulated that AI could reduce care costs for patients in the ICU and improve their prognosis. “There are plenty of areas in critical care where it would be extremely helpful to have efficacious, fair, and transparent AI systems,” notes Gary Weissman, professor of pulmonary and critical care medicine [14]. The full potential of AI will be realized once it becomes a trusted clinical assistant for intensivists. After all, ICUs, which routinely collect a significant volume of data, provide an ideal setting for the deployment of machine learning technologies [15].

### Objectives

Currently, the application of AI in intensive care predominantly focuses on “assisting” health care professionals. This review targeted AI robots in ICUs, primarily discussing relevant advancements in recent years and presenting and analyzing challenges faced in ICUs and potential solutions, as well as strategies for health care professionals to handle AI robotic technologies, for the reference of health care professionals and system developers.

## Methods

### Design

We followed the scoping review methodology proposed by Arksey and O’Malley [16], which includes (1) identifying the research question; (2) identifying relevant studies; (3) study selection; (4) charting the data; and (5) collating, summarizing, and reporting the results. In addition, to ensure the rigor of the scoping review, we adhered to the PRISMA-ScR (Preferred Reporting Items for Systematic Reviews and Meta-Analyses extension for Scoping Reviews) guidelines (Multimedia Appendix 1 [17]).

### Overview

Upon establishing the focus on the application of AI robots in ICUs, we embarked on a keyword search encompassing terms related to robots and intensive care. Due to the novelty and complexity of the research topic, the experimental designs and results presentation vary significantly across the literature, making traditional methods of literature quality assessment inadequate. Consequently, we relied on the profound knowledge and practical experience of domain experts to provide essential perspectives for understanding the complexities presented in the literature. We engaged 4 experts—2 from the IT sector and 2 medical professionals—to devise a search strategy and select appropriate databases. Given the interdisciplinary nature of our research, spanning medicine, robotics engineering, and human-computer interaction design, our search was not confined to medical databases; we also included databases from the engineering field. Our literature search encompassed 3 electronic databases: PubMed, Embase, and the IEEE Xplore Digital Library. We restricted our search to publications in English, imposing no limits on the year of publication. The search was conducted over a brief period, from May 1, 2023, to May 5, 2023. Textbox 1 provides detailed insights into our search methodology.

**Databases and search strings.**

**Search strings**

- PubMed: (“Artificial Intelligence” [Medical Subject Heading (MeSH)] OR “AI” OR “Robotics” [MeSH] OR “Robots”) AND (“Intensive Care Units” [MeSH] OR “ICU” OR “Critical Care Units” OR “CCU”)
- Embase: (“artificial intelligence”/exp OR “ai” OR “robotics”/exp OR “robots”) AND (“intensive care unit”/exp OR “icu” OR “critical care unit” OR “ccu”)
- IEEE Xplore Digital Library: (“Artificial Intelligence” OR “AI” OR “Robotics” OR “Robots”) AND (“Intensive Care Units” OR “ICU” OR “Critical Care Units” OR “CCU”)

**Figure 1:**
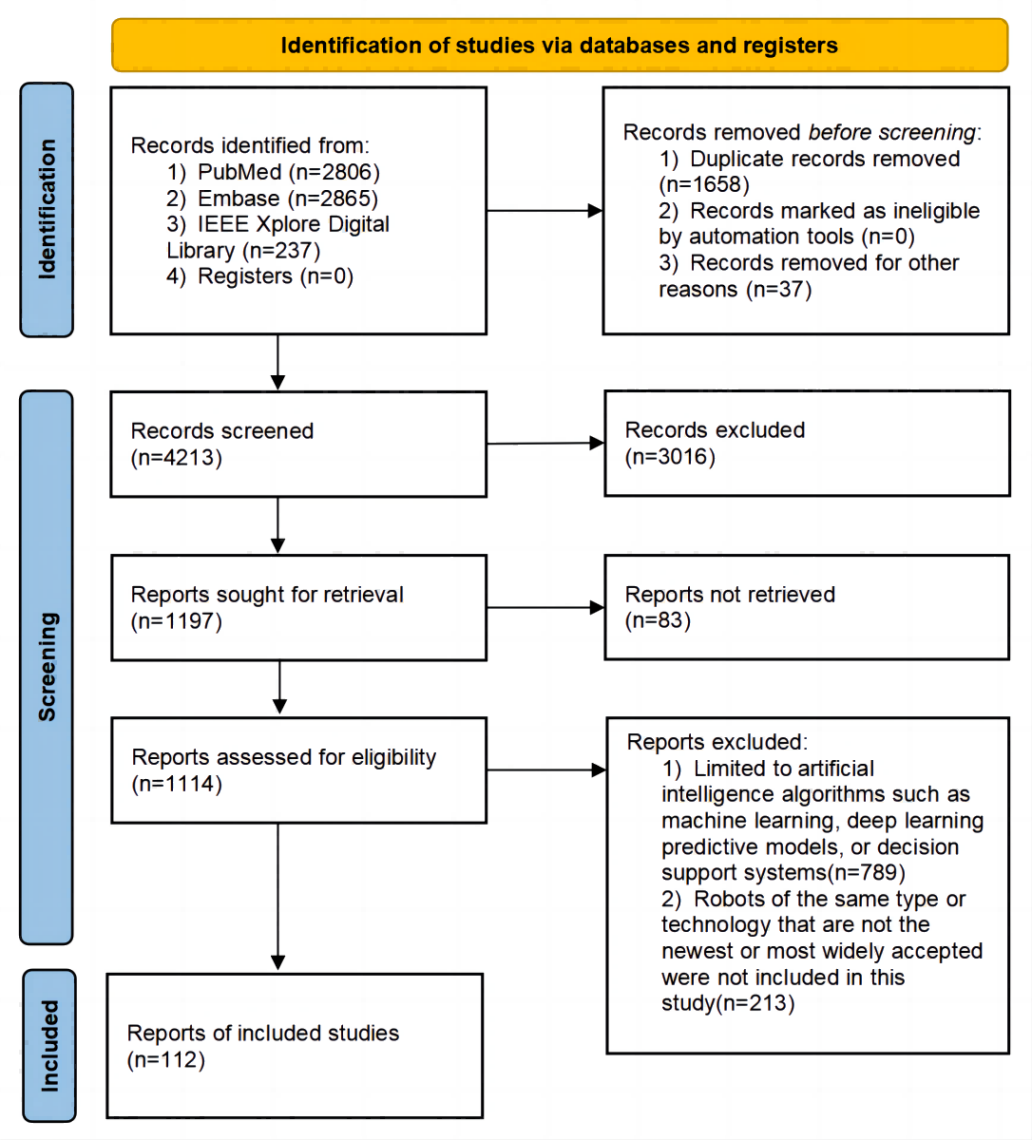
PRISMA (Preferred Reporting Items for Systematic Reviews and Meta-Analyses) flow diagram of the screening process.

During our initial search across the 3 databases, we retrieved a total of 5908 articles. These search results were first imported into EndNote (Clarivate Analytics), a literature management tool, to facilitate the deduplication process. Subsequently, LW and YC independently conducted a screening of titles and abstracts to weed out articles that were clearly irrelevant. Given the market’s saturation with robot products of similar functionalities and the constraints on this review’s length, we opted to focus on the most representative or widely adopted technologies among robots offering the same functions. This selection process led to the inclusion of 77 articles in our review. These studies encompassed multiple phases, including the design, development, and validation of robots, collectively covering 21 different AI robotic products, detailing their development, evolution, and application examples. Figure 1 shows a comprehensive view of our study selection process.

In our examination of the 77 articles selected for inclusion, we meticulously identified the key characteristics of each study. These characteristics encompassed the design purpose of the technology, the AI algorithms used, the anticipated application scenarios, and the target user groups. From this analysis, we developed an initial classification framework aimed at organizing technologies based on their primary functions and application contexts. For instance, technologies designed for nursing tasks were classified under nursing assistance robots, whereas those with therapeutic responsibilities were designated as therapeutic assistance robots. Similarly, devices integrated with ventilators capable of remote operation and achieving therapeutic goals were also classified as therapeutic assistance robots.

To validate the accuracy and logic behind our classification, we sought insights from additional experts in the domains of medicine and information engineering. Their feedback prompted adjustments and refinements to our framework, ensuring that it precisely represented the nuances and relationships among the various technologies.

Subsequently, applying the refined classification framework, we categorized the included studies into their respective groups, deriving organized results. This meticulous classification was undertaken with the aim of ensuring clarity and comprehensibility for all potential readers, including professionals (physicians and engineers), patients, and their families. Our goal was to provide a clear understanding of the applications and potential of various technologies in the ICU, making the information accessible and valuable to a broad audience.

### Inclusion and Exclusion Criteria

This review included studies that met two main criteria: (1) the studies needed to focus on actual products or application cases that conformed to the definition of AI robots, and (2) the application scenario of the study had to be the ICU or any place for patients discharged from the ICU.

We excluded studies that met any of the following conditions: (1) limited to AI algorithms such as machine learning, deep learning predictive models, or decision support systems; and (2) not published in English.

## Results

### Application of AI Robots in Intensive Care

#### Overview

**Figure 2:**
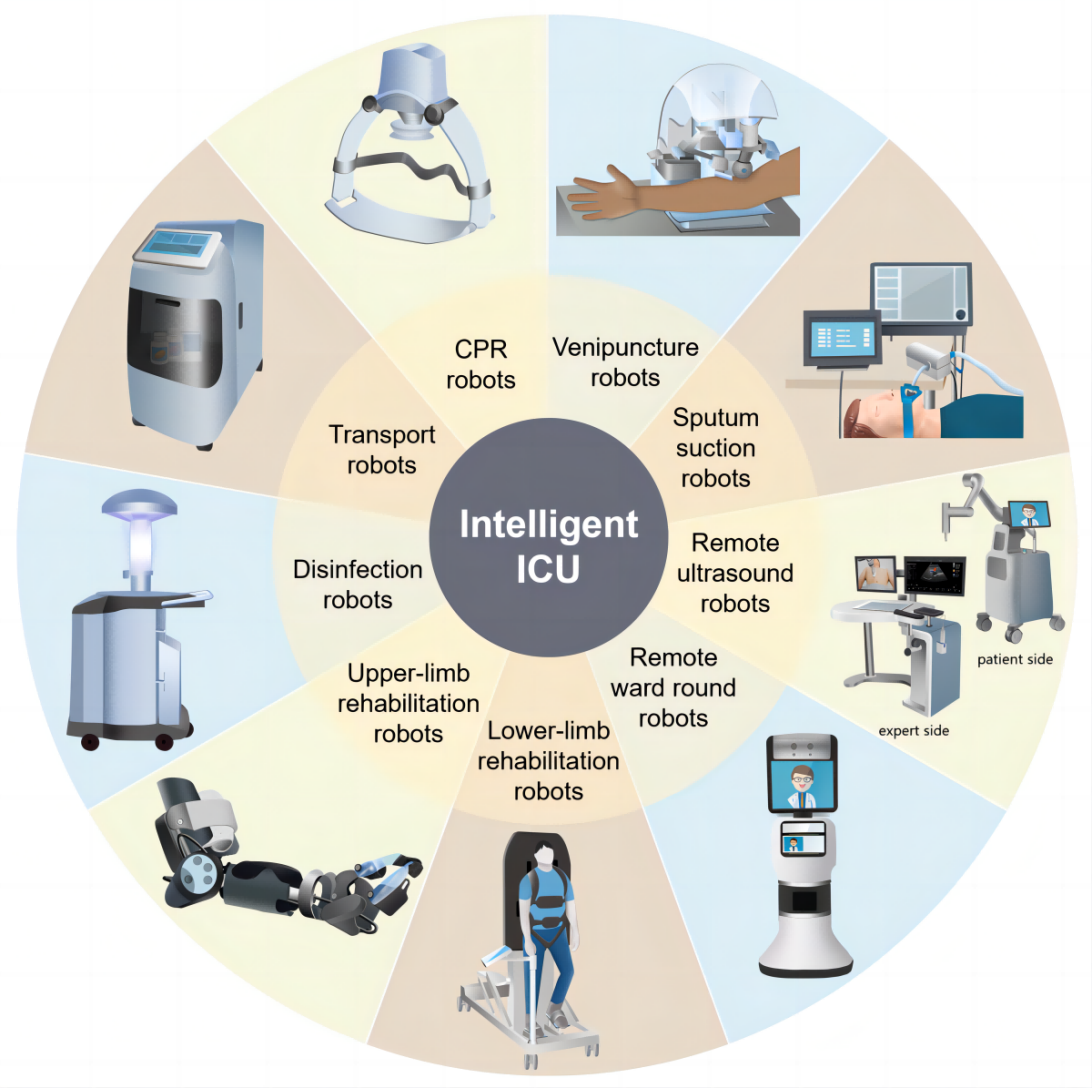
Types of robots available in an intelligent intensive care unit (ICU). CPR: cardiopulmonary resuscitation.

Currently, there is a broad array of experimental studies and AI robots applied in ICUs. We categorized these into 5 main types based on their application scenarios and functions in ICUs: therapeutic assistance robots, nursing assistance robots, rehabilitation assistance robots, telepresence robots, and logistics and disinfection robots (Figure 2). The application of robotics in the medical field is extensive, encompassing myriad functions and scenarios, rendering their precise classification a formidable task. Multimedia Appendix 2 [6,18-90], which is neither exclusive nor exhaustive, summarizes the representative research groups and commercial suppliers to provide readers with a panoramic view of the field.

#### Therapeutic Assistance Robots

The ICU is a specialized department dedicated to the treatment of patients who are critically ill. Driven by the necessity to enhance therapeutic outcomes, mitigate medical risks, alleviate the workload of health care personnel, and provide personalized treatment regimens for patients, therapeutic assistance robots in the ICU have become indispensable [91].

##### Ventilator-Mounted Cartesian Robot

This can also be viewed as a type of telepresence robot. A teleoperated ventilator controller system has been developed, which consists of a custom robotic patient-side device and a touch-based master console. Computer vision tasks that enable an intuitive user interface and accurate robot control are executed on the master console. The system was initially developed and deployed for the most popular touch screen–controlled ventilator at Johns Hopkins Hospital, the Maquet Servo-u (Getinge AB) [18]. Different from the traditional ventilators in most ICUs, the robot performs remote control and monitoring outside the ICU room through the network. The actual ventilator settings are controlled and adjusted remotely via physical controls (such as buttons and knobs) and synchronized real-time image transmission video feedback [19]. It reduces the time staff spend entering the ICU performing simple tasks such as changing ventilator settings, reducing the risk of infection and stress on personal protective clothing resources. Following a qualitative assessment in clinical environments, feedback from respiratory therapists highlighted that the system could significantly empower the respiratory care team by liberating valuable resources. The design’s Cartesian layout enables the robot to approach the operating table in as horizontal a manner as possible. This approach not only minimizes the installation burden but also ensures that the robot’s operation remains closely aligned with conventional manual procedures. Such a configuration facilitates ease of use and integration into existing medical workflows, thereby enhancing efficiency without compromising the quality of patient care [18]. In a simulated ICU environment, a Cartesian robot reduced the total time required for a respiratory therapist to make typical setup adjustments to a traditional ventilator from 271 to 109 seconds, which was 2.49 times faster [18]. More recently, Song et al [20] developed an integrated telemonitoring or operation system with an accurate XY positioner and a 3-df end effector for accurate manipulation (maximum positioning error of 0.695 mm; repeatability is 0.348 mm).

In ICUs where a single brand of ventilator is predominantly used, configuring Cartesian robots proves to be more convenient. Nevertheless, to accommodate a broader array of ICU wards and equipment types such as infusion pumps, it is imperative to expand the variety and range of control interfaces. This expansion aims to enhance the robot system’s capabilities for physical control interactions. However, a significant hurdle to the clinical application of these robotic systems is the challenge associated with their cleaning and disinfection. This issue could be mitigated in the future by enclosing the equipment within acrylic covers, thereby simplifying the process of maintaining hygiene and ensuring the equipment’s safety for patient care [6]. This solution not only addresses infection control concerns but also aids in the seamless integration of robotic systems into the rigorous and cleanliness-focused environment of the ICU.

##### McSleepy

McSleepy (Intelligent Technology in Anesthesia research group laboratory, McGill University, Montreal, Quebec, Canada), a real robot for anesthesia, is able to autonomously control hypnosis, analgesia, and neuromuscular block at the same time with regard to induction, maintenance, and emergence [21]. The anesthesiologist begins by entering patient data, including height, weight, type of surgery, and medication history, on the touch screen. In fully automatic mode, the system will use remifentanil, propofol, and rocuronium or succinylcholine to induce anesthesia. McSleepy assists the anesthesiologist in the same way that automatic transmission assists people when driving. Through a sensor that measures muscle movement, it monitors the patient’s depth of consciousness, pain severity, and muscle movement and injects the corresponding dose of medication intravenously according to a built-in algorithm based on the obtained data. It provides deep or peripheral muscle relaxation in different closed-loop models [22]. As such, anesthesiologists can focus more on other aspects of direct patient care. An additional feature is that the system can communicate with PDAs, making distant monitoring and anesthetic control possible [21,23,24].

In a pilot study in the Department of Anesthesiology and Critical Care of the Bordeaux University Hospital, McSleepy performed automatic anesthesia for cardiac surgery with isoproterenol, remifentanil, and rocuronium without manual control. Automatic cardiac anesthesia was successfully performed in 80% (97.5% CI 53%-95%) of cases. Hypnosis was monitored using the bispectral index, which was <20% of the bispectral index target of 45, showing better control over hypnosis and duration of anesthesia [25]. A randomized controlled trial investigating a novel closed-loop drug delivery system found that automated delivery achieves superior sedation control over manual methods. This improvement is credited to the closed-loop system’s ability to frequently or continuously monitor control variables and adjust drug delivery rates more often, circumventing the fatigue that can impair manual administration [26]. To guarantee the safety of this automated system, numerous protective features have been implemented. These include preventing the administration of muscle relaxants when mask ventilation proves difficult and querying any manual actions that breach established safety protocols [27]. Recent advancements in research highlight the successful application of robotic technology in anesthesiology. Beyond the surgical anesthesia applications that we have detailed, delicate procedures such as endotracheal intubation are also seeing promising developments [28]. Anesthesiologists are encouraged to engage with robotic systems, leveraging their significant contributions to enhancing medical quality and efficacy, thereby ensuring the highest standard of patient care and treatment.

##### Cardiopulmonary Resuscitation Robots (Seoul University Medical College, South Korea)

Some reports indicate that failure of cardiopulmonary resuscitation (CPR) is an important limiting factor for life-prolonging treatment of patients in the ICU [29,30]. Robots have enough power to achieve high-quality CPR, which could overcome the shortcomings of manual CPR and mechanical CPR devices. Recently, Jung et al [31] developed an automated robotic CPR system that performs CPR automatically, analyzes the patient’s condition, and relays the information to the CPR system. In the initial state of the CPR process, the robot manipulator determines the optimal compression position by adjusting the point of pressure and, guided by end-tidal carbon dioxide levels, periodically and repeatedly delivers adequate speed and depth for performing CPR. The combined CPR system can accurately capture the compression condition of patients, overcome the blank periods when medical personnel alternately perform CPR, and increase the probability of cardiac resuscitation [31,32].

In a study using a porcine model of cardiac arrest, the use of a CPR robot did not significantly enhance the success rate of resuscitation efforts compared to manual CPR. However, there was a notable improvement in the neurological deficit scores observed 48 hours after resuscitation, suggesting a potential benefit in postresuscitation neurological outcomes [32]. Another study indicated that robot-assisted CPR outperformed traditional manual CPR methods, achieving higher resuscitation success rates in patients without specific injuries [31]. The anticipated benefits of incorporating robot-assisted CPR into clinical settings extend beyond merely improving resuscitation success rates. They also include the potential to reduce labor costs and minimize the instances of ICU staff congregating around a patient’s bedside to alternate performing CPR. This technological advancement suggests a promising direction for enhancing the efficiency and effectiveness of resuscitation efforts, thereby improving patient outcomes while optimizing resource use within critical care environments.

#### Nursing Assistance Robots

Nursing care robots, designed to assist patients who are bed-ridden with simple long-term care services, have been widely used in hospitals to assist older adults and individuals with disabilities. The many invasive diagnostic procedures and heavy nursing workload (multiple multitasking) in the ICU make medical staff vulnerable and overloaded, with very limited ability to provide timely care to patients. Through the intervention of intelligent robots, heavy physical activity in nursing work is alleviated to a certain extent, and nurses are liberated from it so that they can put their energies into more professional and meticulous nursing work.

First, despite the proliferation of devices currently available on the market to assist with venipuncture, there remain certain limitations. Technologies such as ultrasound, which can easily penetrate human tissue, thereby enabling the high-resolution visualization of both shallow and deep tissue structures for vascular guidance, and devices such as the VeinViewer and AccuVein AV300 [33,34], which use near-infrared spectroscopy technology for vascular imaging, are fairly mature. However, these techniques are still constrained by their inability to provide depth of the vein as well as the fact that imaging technology does not directly aid the insertion of the needle. VenousPro (VascuLogic, LLC), an automated robotic venipuncture device, addresses these issues. It identifies vessels suitable for cannulation and robotically guides an attached needle toward the lumen center, enabling the safe drawing of blood from peripheral forearm veins [35,36]. The device uses 940-nm near-infrared light to enhance the contrast of the subcutaneous peripheral vein, selecting the appropriate vein through real-time imaging and mapping of the 3D spatial coordinates of the subcutaneous vein [36]. Chen et al [36] evaluated the system’s cannulation accuracy on a vascular model through tracking, free-space localization, and use of a dark-skinned phlebotomy training model. Early versions of the device successfully demonstrated the feasibility of automatic venous access with a 100% success rate for venipuncture and placing of the needle in the desired position with high precision (mean positioning error 0.21 mm, SD 0.02 mm) [35]. This effectively reduced the risk of acupuncture injury [37].

Subsequently, the team developed an improved instrument based on a 9-df image-guided venipuncture robot, which increased radial rotation in response to rolling vein deformation and allowed for real-time adjustments to the pose and orientation of the needle [38]. Through robust optimization of near-infrared imaging coupled with image analysis and a robotic control system, this venipuncture robot realizes the automation of venipuncture, minimizing the likelihood of needlestick injuries and related bloodborne infections. This not only liberates nursing staff from the repetitive task of venipuncture but also protects the safety of practitioners [36]. In addition, this device may, in the future, be integrated with diagnostics, facilitating automated blood draws and rapid patient information synchronization. Moreover, the foundational imaging, computer vision, model recognition, and robotic technology developed for this device can be extended to arterial pathways, computer-assisted diagnostics, and miniature robotic surgery, potentially revolutionizing practice in ICUs and other departments.

Second, prompt and effective airway management can prove pivotal to the survival of patients who are critically ill. However, for patients with substantial secretions, sputum aspiration undertaken by nursing staff becomes a frequent necessity, significantly demanding the latter’s time and effort. Therefore, a sputum suction robot (Xi’an Jiaotong University, China) has been developed, which is a simple 6-df manipulator with a steering gear. Exhibiting stable movement, the sputum suction robot is proficient in smoothly performing the clamping, insertion, back-off protection, and removal of the suction tube, thereby achieving effective sputum aspiration [39]. Naturally, the current iteration of this sputum suction robot faces certain challenges, such as the complexity of the mechanical arm structure and limited mobility that may result in some sputum being left unaspirated. The simplification of the driving mechanism and the optimization of mobility present promising directions for future enhancements. Such improvements aim to realize high-precision, high-quality automated sputum suction within the ICU.

Third, kangaroo care is a nursing method aimed at premature infants. It serves as a nonpharmacological intervention for treating surgical pain in infants. By promoting skin-to-skin contact between the infant and parents, it reduces the adverse effects of repeated surgical pain on the long-term development of the nervous system [40]. However, a notable challenge within the neonatal ICU (NICU) setting is that parents cannot always be physically present to provide this essential care. This limitation calls for innovative solutions to ensure that premature infants still receive the benefits of kangaroo care, possibly through alternative methods or support systems that can simulate the presence and therapeutic effects of parental contact. Calmer (University of British Columbia, Vancouver, British Columbia, Canada) was developed to manage acute pain effectively for preterm infants in the NICU by simulating key pain-reducing components of human touch–based treatment. The human tactile pain treatment effect is achieved by placing the infant prone on the Calmer, which provides sensory intervention similar to parental skin contact while simulating the heartbeat and respiratory frequency of the parents with customized physiological signal processing software to mitigate the adverse neurodevelopmental effects of early pain exposure in preterm infants [41]. The effects of this robotic device on pain management during routine blood collection were studied in 10 infants. Calmer reduced physiological pain reactivity during and after painful blood collection procedures [34,40]. This approach not only aligns with the philosophy of nonpharmacological pain management but also potentially saves the NICU approximately US $380,000 per year in nursing time (United States). However, it is crucial to clarify that Calmer does not intend to replace the role of parents or neonatal care. It merely provides an alternative solution when such avenues are not available.

Fourth, older patients consistently represent a predominant segment of ICU admissions, manifesting unique characteristics and frequently presenting with cognitive impairments and mobility limitations. Physical assistive robots are strategically engineered to address the exigencies of daily living activities [92]. A robot named Paro has been extensively integrated within ICU environments and subsequent post-ICU discharge home care, offering socioemotional and psychological support to older patients in the ICU. With its lifelike appearance and behavior, Paro emulates genuine animal esthetics and movements, enabling it to perceive and respond to human vocalizations, touch, and actions. Recognized as one of the world’s premier nursing assistance robots, Paro has been commercialized and operationalized across diverse care settings, particularly within ICU care, in multiple countries [42]. While it is designed to provide cognitive stimulation, no definitive evidence has surfaced indicating immediate or long-term cognitive improvement after Paro intervention. However, we cannot overlook its positive psychological impact in several settings. Its widespread application and accessibility also serve as a blueprint for the development of companion robots [43].

Beyond the 4 nursing assistance robots previously mentioned, a diverse range of technologies is under development aimed at enhancing nursing practice. These technologies are designed to assist with various tasks, including feeding patients, transporting patients, bathing, providing emotional support, administering medication, and facilitating other daily activities. Notable examples include the Care-O-bot, a compact, highly integrated service robot that primarily functions as a household assistant [44]. Another example is “RI-MAN,” a soft-bodied robot designed to safely lift adults [45]. While these robots undertake certain nursing responsibilities and are potentially beneficial to the general population, they were not included in the scope of this review. This decision reflects our focus on specific categories of medical robot modules directly applicable to critical care and ICU settings, thereby delineating a clear boundary for our review’s content and objectives.

The extensive research by Locsin [93] underscores a future where the integration of nursing with AI technology is not just a possibility but an inevitability. This projection implies that, as technology evolves, nurses will need to become proficient in advanced technological tools, ensuring that their participation in clinical practices is grounded in informed consent. In the nursing practices of the future, technology will serve to augment human nursing activities. This includes leveraging technology for predictive interventions to enhance nursing efficiency. However, it is critical to note that human cognition and emotional intelligence will remain at the heart of technological care. This integration suggests a model of care where technology and human elements complement each other, ensuring that patient care remains personalized, empathetic, and efficient, thereby reflecting the intrinsic values of nursing while embracing the advancements of AI technology.

Thus, the future of nursing practice is envisioned as a symbiotic relationship between human nurses and medical robots. This partnership aims not merely at task completion but also at leveraging sophisticated technology to deepen patient understanding and enhance care, thereby elevating the standard of nursing services provided. Human nurses are poised to maintain their pivotal role in health care, with person-centered care continuing to serve as the foundational principle of nursing. Through this fusion of technology and human touch, nursing will evolve, ensuring that care remains empathetic, responsive, and fundamentally human at its core.

#### Rehabilitation Assistance Robots

Most patients who are critically ill, confined to bed rest for extended periods, exhibit a high incidence of ICU-acquired weakness, significantly influencing patient prognoses [94]. Rehabilitation therapy, a potential solution to this issue, can notably enhance patient outcomes and quality of life. Numerous studies have attested to the pivotal role of early rehabilitation in improving patient prognoses and ensuring quality of life. Rehabilitation robots are devised to aid the restoration of impaired sensory, motor, and cognitive skills [95]. The deployment of these robots can relieve physicians of strenuous training tasks, allowing for the analysis of robot-derived data during training sessions to evaluate patient rehabilitation status. It has been established that intensive locomotor training can affect improvements in walking function for patients with movement disorders after stroke or spinal cord injury (SCI) [96-98]. Rehabilitation robots facilitate more extensive and intensive training compared to traditional therapeutic methods. Moreover, rehabilitation robots typically engage and challenge patients to meet objectives interactively. Although their application within the ICU is currently minimal, these robots hold promising potential in assisting early rehabilitation efforts for patients in the ICU. There are presently several commercially available and experimental-stage noncommercial rehabilitation robots reported in the literature. Some cases are presented in the following paragraphs.

First, Lokomat (Hocoma, Inc) is a sophisticated robotic system designed for gait rehabilitation. It targets patients who exhibit locomotor anomalies induced by brain injury, spinal cord damage, neurological disorders, muscular injuries, and orthopedic diseases, facilitating improved mobility in patients who are neurologically compromised [46,47]. The Lokomat system comprises a structural frame affixed to a treadmill, featuring a load-bearing arrangement such as a gait orthosis. When attached to the patient, it modulates hip and knee joint movements to generate a predefined gait pattern [48]. Regular Lokomat-assisted training has been effectively used in rehabilitating patients who were comatose with cerebral hemorrhage. Following a 4-month therapeutic regimen, patients exhibited remarkable improvements in the severity of their comatose state (as per the Glasgow Coma Scale score of 4), an extension in walking duration from 15 to 32 minutes, and recuperation of eye and joint movement [49].

In a rigorous evaluation, Chillura et al [47] used Lokomat in a 6-month intensive conventional rehabilitation therapy. The outcome was a marked improvement in muscle strength (42/60), physical and mental independence (80/126), and 6-minute walk distance (47 m) in patients in the ICU with acquired weakness. A separate prospective study corroborates the potential of Lokomat in ameliorating patient rehabilitation after stroke [50]. A randomized single-blind, parallel-group clinical trial (40 participants per group) showed that training with the Lokomat system for 3 to 6 months after lesion can improve the walking ability of patients with incomplete SCI. This improvement was manifested in increased walking endurance and enhanced strength in the lower limbs [51]. Furthermore, 6 additional randomized controlled trials using the Lokomat system corroborated these findings [52-57], underscoring the consistency and reliability of the Lokomat as an effective rehabilitation tool for improving mobility and strength in individuals with incomplete SCI. This body of evidence strongly supports the Lokomat system’s role in advancing the recovery process for patients with SCI, offering them a viable pathway to regain mobility and improve their quality of life.

Currently, advancements in robot-assisted lower-limb rehabilitation have led to the development of 3 main types of devices: exoskeleton, end effector, and portable powered robotic exoskeletons. End-effector devices interact directly with the patient’s lower limbs, applying force, assisting movement, or guiding patients through specific movement patterns, exemplified by the “G-EO-Systems” [99] and “Haptic Walker” [100]. Exoskeleton-type devices, exemplified by “LOPES” from Delft University of Technology in the Netherlands [101], encase the patient’s legs and provide support and movement assistance directly aligned with the limb’s natural biomechanics. Among these, Lokomat stands out due to its superior customization capabilities, safety, comfort, and integration of virtual reality (VR). It also boasts features such as participation detection and motivational elements to engage users more effectively in their rehabilitation process. With 651 institutions worldwide adopting Lokomat, its widespread use underscores its significant advantages and effectiveness in facilitating the rehabilitation of patients with lower-limb impairments [58].

Such findings hint toward a broad applicability of Lokomat in ICU settings, enabling both early and post-ICU rehabilitation to be personalized and intelligent. As AI and machine learning continue to advance, the Lokomat system’s ability to analyze patients’ gait data and physiological indicators to offer precise rehabilitation strategies and real-time feedback is becoming a reality. Leveraging VR technology may render the rehabilitation training environment for patients in the ICU more captivating and web-based, fostering increased patient engagement and positivity during the recuperative process. Furthermore, integration with biometric sensors and remote monitoring technologies could feasibly enable real-time tracking and remote guidance of patients’ rehabilitation progress, heralding the future direction of Lokomat’s potential applications.

Second, aside from lower-limb or gait assistance rehabilitation robots, research into upper-limb rehabilitation robotics remains an active field of investigation. ArmeoSpring (Hocoma, Inc) is a rehabilitative exoskeleton that stabilizes the arm via a fixed frame. It is a passive exoskeleton that uses adjustable springs to deliver gravitational compensation for the patient, thus allowing the individual undergoing rehabilitation to concentrate solely on executing requisite tasks. Despite its passive nature, the ArmeoSpring exoskeleton is laden with sensors, enabling its application as an evaluation instrument for assessing the capacity and range of the user’s arm [59]. These sensors also facilitate interactive training and integration with VR, enabling patients to simulate task-oriented motor exercises within a virtual learning environment on a computer screen, providing auditory and visual performance feedback during and after interaction.

A 5-year randomized controlled trial involving a subacute stroke program evaluated 215 patients with stroke with moderate to severe arm injuries who were undergoing rehabilitation therapy [60]. It appeared that there was no substantial difference in functional upper-extremity improvement with ArmeoSpring robotic intervention, although sensorimotor scores did demonstrate enhancement (13.32 vs 11.78). This equivocal result could be ascribed to a lack of sequencing of early testing interventions [61]. Conversely, another clinical trial involving a patient with mild to moderate hemiparesis demonstrated significant improvement in upper-extremity arm motion after 4 months of ArmeoSpring treatment [62]. It remains uncertain whether robotic-assisted rehabilitation definitively surpasses conventional physical therapy; rather, it appears to offer advantages to traditional treatments. In addition, in contrast to conventional physical therapy, careful patient selection is essential for robotic-assisted interventions. Factors such as the degree of functional impairment, age, disease duration, and cognitive levels play significant roles in this selection process. A study focusing on the evaluation of upper-limb movement parameters in patients after a stroke using the ArmeoSpring demonstrated its effectiveness in reliably and sensitively assessing motor impairments and the influence of therapeutic interventions on the motor learning process. The research highlighted the device’s potential as a valuable assessment tool for quantifying sensorimotor disorders in the upper limb [59].

Another type of end-effector robot, the “MIT-MANUS” (Massachusetts Institute of Technology, United States) [63], trains the upper limb by applying force at a single point on the patient’s arm. However, due to its linkage mechanism, the robot has a long mechanical arm and low torque output capacity, making it difficult to perform tasks requiring high-load resistance training. In addition, it has a large footprint. It lacks the ability to perform complex movements of the upper-limb joints, similar to the ArmeoSpring. Therefore, we did not include the MIT-MANUS in this review.

Third, patients in the ICU not only grapple with their primary illness but also frequently experience newly acquired long-term physical, psychological, and cognitive impairments, collectively referred to as post–intensive care syndrome [102]. Given the inconsistency in results from various screening tools, there is an urgent need for an objective, comprehensive, simplified, and unified assessment tool. The Kinesiological Instrument for Normal and Altered Reaching Movements (KINARM; Kingston, Ontario, Canada) is a robotic research tool specifically designed to execute quantitative neurological evaluations of sensorimotor, proprioceptive, and cognitive brain functions. It comprises a wheelchair and an upper-extremity exoskeleton tailored to patients based on their physical specifications. The KINARM permits researchers to gauge the coordination of limbs across multiple joints while also precisely measuring the joint-specific force exerted by the patient during task execution. The precision of this tool eliminates the subjectivity typically intrinsic to physiotherapeutic assessments of neurological status, such as muscle tone, spasticity, proprioception, and others [64].

A total of 104 patients in the ICU underwent sensorimotor and neurocognitive assessments using the KINARM 3 and 12 months after discharge. The team then performed a series of kinase evaluations on stroke survivors 3 and 12 months after discharge in patients who were critically ill and receiving acute renal replacement therapy and obtained a 0.3 correlation (90% strength in 89 patients) between regional cerebral oxygen saturation (a surrogate marker of cerebral autonomic regulation) and delirium in patients who were critically ill through KINARM scoring [65]. The tool has also been used to assess the correlation between brain tissue oxygenation (a surrogate marker of brain perfusion) during the acute phase of critical illness (ie, 24 hours) and long-term neurological dysfunction [66,103], as well as to evaluate sensorimotor deficits in patients with stroke and traumatic brain injury [67,104]. The KINARM provides objective and quantifiable data for sensorimotor and neurocognitive functions in ICU survivors. As a diagnostic and assessment tool, it aids rehabilitation and supports ICU survivors in regaining autonomy and independence in their daily lives. Despite its limitations, efforts must continue to enhance mobility and portability, broaden applicability and scope, and reduce cost and operational complexity.

Compared to traditional physiotherapy, robot-assisted rehabilitation based on AI and VR offers patients more intensive, systematic, repetitive, and task-oriented rehabilitation training, which plays a crucial role in promoting the process of functional recovery. Although many studies have shown that robot-assisted rehabilitation can effectively enhance the rehabilitation effect, a review of the clinical application of stroke rehabilitation points out that robot-assisted therapy has not shown obvious advantages in improving the motor function of patients with stroke [68]. Compared with traditional training or stand-alone training, its effect on the rehabilitation of patients with chronic stroke is still questionable. Similarly, the effectiveness of using exoskeleton devices for upper-limb motor function training also lacks sufficient evidence [105]. Therefore, at the current stage, robot-assisted rehabilitation therapy should be considered as a supplement to traditional physiotherapy, not a replacement. There is also no clear evidence to show that robot gait training can outperform traditional physical therapy when applied alone to patients with chronic stroke [106]. On the basis of the existing evidence, we can conclude that robot-assisted rehabilitation therapy can improve the motor function of patients needing rehabilitation and serve as an additional treatment intervention in combination with traditional rehabilitation therapy. However, with the further development of AI and machine learning technology in the future, we expect robot-assisted rehabilitation therapy to have greater development potential.

On the other hand, we must acknowledge that the current rehabilitative assistance robots face 3 core challenges: energy endurance, comfort assurance, and cost control. First, we need to change the existing endurance mode and adopt more effective energy supply methods. Second, we need to solve the comfort issues that may arise during the use of robots, such as blood circulation problems and muscle deformation that may be caused by wearing methods. Finally, we need to focus on controlling costs so that all patients who need rehabilitation can afford it.

#### Telepresence Robots

Telepresence represents a potential avenue for enhancing information accessibility for providers, encompassing aspects such as patients’ visual and auditory feedback, bedside care, and vital sign data facilitated by remote monitoring or telechecking [107]. Given that most patients in the ICU are susceptible to unpredictable conditions, there is an acute need for swift identification and prompt response during emergent situations. The significance of telepresence robots lies in their ability to deliver expert health care services over distances, effectively mitigating the need for colocation of physicians and patients. This approach greatly augments the accessibility of health care services for patients in remote areas. Moreover, it potentially eradicates the likelihood of infectious disease transmission between patients and health care professionals [108]. Using AI and human-machine interaction, these telepresence robots supplement diagnostic and therapeutic processes via medical professionals’ expertise, thereby enhancing the exchange of visual and electronic information between the patient and health care staff [109]. To illustrate this, we provide the following example.

InTouch Health Remote Presence-7 (RP-7; InTouch Health Systems) is a real-time audiovisual robotic telepresence system that provides communication among patients, hospital staff, and remote physicians [33,69,110]. Remote assessors used the RP-7 robot end point to conduct their clinical coma evaluations [69]. Compared with the total scores on the Glasgow Coma Scale or Full Outline of Unresponsiveness of the remote physician evaluators, the RP-7 robotic system had a similar score (difference in scores of 0.25 and 0.40, respectively), and it can serve as a reliable scoring system to help evaluate patients in a coma [69]. In an ICU setting, the RP-7 assisted in the assessment of increased efficiency, care coordination, and throughput, with a decrease in patient ICU stay (–0.8 days), an increase in hospital discharges (+11%), and a significant decrease in the number of unexpected events (–1.2 days) [70,109]. Regarding the team cooperation ability (attitude, behavior, and cognition) of clinicians, the use of the RP-7 maintained the cooperation, trust, communication, and psychological safety of the team [107]. However, the RP-7 does not enhance collaboration between nurses and physicians in patient care decisions as compared to traditional telephone night checks [111].

A year later, the InTouch Vita, developed by InTouch Health and iRobot, was found to improve the independence of remote clinicians in managing patient care [71,112,113]. The Vita has an improved navigation system with an autopilot feature that enables remote service providers to directly control or automatically direct it to a predetermined location for improved efficiency [113]. In addition, the product provides real-time clinical access to patient data and has been cleared by the Food and Drug Administration for active patient monitoring that may be needed for immediate clinical action [72]. The Remote Presence Virtual + Independent Telemedicine Assistant (RP-VITA) also has an iPad interface that allows pilots to browse quickly and easily [114]. Unlike the RP-7, which must be powered using an active joystick, the RP-VITA only needs a mobile phone to log into the system and issue commands verbally to move the robot around to the designated location to complete the task [71]. To enable the robot to accurately follow the target character and maintain the corresponding safe distance and speed, Long et al [73] used the improved Gaussian filter algorithm to estimate and correct the centroid of the human body in real time, effectively improving the stability and safety of human tracking.

As a remote, virtual, and independent telemedicine assistant, the RP-VITA enables physicians to directly interact with patients from anywhere in the world. This technological advancement effectively transcends the traditional barriers of physical and biological constraints commonly encountered in ICU settings, where the immediacy of medical services is crucial yet often challenging to maintain. With the RP-VITA, physicians no longer need to undertake urgent commutes to the ward; instead, they can simply access the robot platform from the comfort of their home, offering a solution that is not only more expedient but also significantly more convenient [74]. This achieves truly meaningful health care services, delivering the right expertise to the right place at the right time to do the right thing at the right price. The RP-VITA exemplifies the future of health care delivery, emphasizing efficiency, accessibility, and the strategic allocation of medical expertise.

Second, Stevie has a stethoscope port and a high-definition pan-tilt-zoom camera, which can relay information during an examination of a patient and help physicians identify illnesses and diseases in the ICU [75]. It was developed by a research team from Trinity College, Dublin, Ireland, and is currently in use at Steve Biko Academic Hospital, South Africa, in the second design iteration [76]. It was designed to be neutral so as to avoid perceptions of gender, race, and age [115]. The upper body of the robot consists of a humanoid head (digital display), trunk, and 2 short arms [116]. A humanoid form makes robots more acceptable [117]. The control system gives the user the ability to operate the robot remotely, including controlling the robot voice and media volume as well as motion and motion. In addition, Stevie’s humanoid facial expressions convey clear emotions [76]. Humanlike limbs can be used to form intuitive gestures, emphasize emotional states, and direct attention, conveying more information than facial expressions [116]. To provide static stability, the robot is equipped with wheels that support omnidirectional, allowing it to maneuver smoothly in any direction without shifting its base. This feature is essential for navigating complex environments and enhancing operational efficiency. Stevie is known as the “most popular baby” for the ICU team [115].

Third, MGI Ultrasound System-Remote 3 (MGIUS-R3; MGI Tech Co, Ltd), a robot-assisted teleultrasound diagnostic system, has considerable application value in the ICU [77,118]. It combines a robotic arm, an ultrasound imaging system, and audiovisual communication for remote manipulation, allowing physicians to manipulate the robotic arm and adjust parameters outside the ICU room for remote ultrasound workups via the fifth-generation (5G) network technology for real-time transmission of audio, video, and ultrasound images [78].

It has been used as a long-range ultrasound device to fight the COVID-19 outbreak in many places in China such as Wuhan [79]. Powerful cloud data transfer rates (up to 10 GB per second) with management mode enable high-definition image capture and high-quality information transfer for large data set sharing. At a distance of 700 or even 1479 km (between the isolation ward of Zhejiang Provincial People’s Hospital and Central South Hospital of Wuhan University in Hubei Province and between Wuhan city in Hubei Province and Sanya city in Hainan Province), it can successfully conduct remote ultrasound examination of the lung and other parts of the patient with the quality meeting the requirements for clinical diagnosis [80,119]. A double-blind diagnostic trial of COVID-19 in 22 cases showed that the diagnostic accuracy of the MGIUS-R3 ultrasound for positive lesions was good (93%), which could replace the traditional scanning method to some extent (difference: *P*=.09) [77]. Another study also successfully performed ultrasound examination of the liver, gallbladder, pancreas, spleen, and kidney in 32 patients, and the high-quality image (average score of 4.73) met the requirements for remote ultrasound diagnosis [78]. Overall, the MGIUS-R3 is noninvasive and repeatable, reduces the risk of infection for patients and physicians, increases safety, and is highly feasible in the ICU. However, all remote ultrasound procedures and communications for the robot are based on 5G networks, which require stable network support throughout the procedure [119,120].

Through the network, remote ultrasound robots transmit ultrasound images of challenging cases from remote areas to tertiary hospitals, where consulting experts diagnose and analyze them, providing decision feedback. This provides an effective solution for situations with limited medical resources, lack of expertise, and high risk of infection.

Fourth, the intrinsic characteristics of the ICU, despite their provision of the highest level of medical care and reliance on advanced organ-support therapies, inherently limit the ability to facilitate real-time visits and communication between patients and their family members. This restriction not only affects the emotional well-being of patients due to a lack of verbal encouragement and emotional support from loved ones but also poses a challenge to patient recovery processes within the high-stress environment of the ICU. For the safety and emotional needs of patients and their families, as well as to achieve the goal of resource optimization, visiting robots have emerged. In 2021, the first 5G+ medical robot+VR visiting system was officially launched in the stroke ICU ward of West China Hospital of Sichuan University. The system successfully enables remote visits for family members at designated hospital locations equipped with matching VR glasses, allowing family members to interact with patients in the ICU in real-time through 2-way communication, facilitating an immersive visitation experience at the bedside [121]. The family members only need to wear VR glasses in the designated place of the hospital and operate the robot remotely through a computer, iPad, or mobile phone. The robot will automatically go to the patient bed designated to be visited after receiving the instruction. 5G networks with high-speed and low-latency transmission features (speeds of 10 to 30 Gbps) transmit back real-time 8K (resolutions of up to 7680 × 4320 pixels) ultra-high-definition full-motion video, enabling families to “physically” visit a patient’s bedside [81,121].

The introduction of visiting robots represents a significant step forward in addressing a previously underappreciated aspect of health care—the impact of patient relationships, particularly with family members, on the process of disease recovery. By facilitating connections that were once hindered due to logistical, health, or institutional barriers, these robots enhance the humanization of health care services. They embody an innovative shift in the delivery models of health care services, ensuring that emotional support and the therapeutic benefits of family presence are not overlooked in patient care. This innovation underscores the importance of integrating technological advancements with the core values of empathy, care, and support, thereby enriching the patient experience.

#### Logistics and Disinfection Robots

##### Overview

The number of medical interventions in ICUs is larger than that in the general ward, and they are more invasive. In addition, the physiological condition of patients who are critical is often fragile, which makes patients in the ICU particularly vulnerable to iatrogenic injury [122,123]. At the same time, ICU health care workers face a high risk of infection [124]. The presence of logistics and disinfection robots avoids the spread of viruses, ensures clean areas for clinicians and patients, and minimizes the risk of infection for medical staff. Logistics and disinfection robots could have also worked in this application in a hospital setting during the COVID-19 pandemic, focusing on these same tasks and supporting patient management [125]. The common forms of logistics and disinfection robots are transport robots and infection control robots, which can solve the problem of ICU staff shortages and share heavy and tedious work. A brief description follows.

##### Transport Robot HelpMate (HelpMate Robotics Inc)

The transportation of goods and patients is an indispensable part of intensive care. However, a large amount of reciprocating work will consume most of the physical strength of nurses, long-term handling of patients will also cause serious lumbar muscle injury in nurses, and unarmed handling may cause secondary injuries to patients. HelpMate is a mature solution that provides autonomous transportation of materials and supplies, with users dispatching tasks through their console interface, and autonomous operations with unsupervised navigation technologies, such as proximity sensors for obstacle avoidance and path planning for navigation [48]. In the ICU, the use of automated transport vehicles will increase efficiency and avoid the potential for cross-infection, especially during the COVID-19 lockdown [82].

As early as the 1980s, the object transfer robot HelpMate was used to carry medical supplies, meals, and experimental samples, among other things. HelpMate mainly navigates based on prestored maps and has a certain obstacle avoidance ability [83]. In 2003, the University of Maryland Medical Center began a pilot program to determine the logistical capabilities and functional utility of the automated pharmacy system II Robot-Rx (McKesson) robotic technology in the delivery of medications from satellite pharmacies to ICU patient care units [84].

In view of the situation that it is not suitable to change a patient’s posture during the process of transfer, Osaka General Medical Center in Japan found that more than half of nurses were willing to use a robot for patient transfer after using the robot Coupling-Parallel Adaption Merged (bilateral transfer bed, mainly through a conveyor belt to complete patient transfer between the bed and stretcher), and nearly half of the patients showed no discomfort during transfer [85].

##### Infection Control Robots

Infection control robots could make ICU wards safer and cleaner than ever before [126,127]. They are a major step up from traditional human cleaning methods, which take more time, are less effective, and often miss crevices that can hide nasty bugs. This is especially important for ICUs and clean rooms for patients who are immunocompromised, and it is possible that automated cleaning will soon become standard practice throughout the health care system. The existing disinfection robots can be mainly divided into 2 groups: UV robots and hydrogen peroxide vapor robots.

The Xenex robots use pulsed xenon to create intense bursts of broad-spectrum UV light that can cut bacterial contamination by a factor of 20 and kill 95% of deadly pathogens. More than 100 hospitals now use Xenex robots [86].

An “EPS” logistics disinfection robot (Ipsen Smart Health Tech [Shenzhen] Co, Ltd), which has both transport and disinfection functions, has been officially on duty in the ICU of Central South Hospital (Wuhan, China), undertaking the drug distribution work of nurses from the station to the ICU wards of patients with severe COVID-19 [87]. At the same time, it can customize the disinfection time and route for high-frequency areas of physician-patient activity and can carry out automatic UV disinfection.

The hydrogen peroxide vapor disinfecting robot combines a hydrogen peroxide device with a robot (Shanghai Jiao Tong University and Lingzhi Technology joint research and development, China) [88]. The disinfecting system inside the robot generates disinfecting gas and can realize autonomous navigation and autonomous movement in an unmanned environment. At present, this disinfection robot has been effectively used in the prevention and control of the COVID-19 pandemic [89]. It is mainly used in ICUs, negative pressure isolation wards, infectious wards, and other closed spaces requiring sterilization.

Moreover, the literature has reported a novel robot system capable of real-time air pathogen monitoring in ICUs. The system comprises an automatic guided vehicle, an air sample collector, and a pathogen detection system. By autonomously patrolling and collecting air samples, the robot uses biosensor technology to perform real-time detection of airborne pathogens. This detection process includes lysis of pathogen particles, amplification of target sequences, and sensitive detection via the Clustered Regularly Interspaced Short Palindromic Repeats (CRISPR) associate system. This robot system allows for real-time monitoring of airborne pathogens within the ICU, aiding in the prevention of hospital-acquired infections and reducing the burden of ICU management. Endowed with a high degree of autonomy and real-time responsiveness, this system provides a new avenue for infection control within ICUs [90].

The ICU is a highly specific and complex area for monitoring patients who are critically ill, requiring not only 24-hour continuous care but also more intensive, timely, and coordinated interventions. Although the introduction of AI robots into ICUs is still in the initial stage, their autonomy, ease of training, and strong adaptability enhance their performance and provide substantial assistance to medical staff. In particular, the COVID-19 outbreak has increased the demand for AI robots that are not fatigued or infected, which may reduce the fatigue of ICU medical staff, reduce medical errors, and improve patient safety. However, for example, monitoring robots for single-organ life-support devices, comprehensive cardiopulmonary-monitoring robots for patients with multiple organ failure, and robots that are flexible in dealing with various unexpected tasks arising during patient care have not yet appeared. Therefore, the demand and development potential of AI robots in ICUs is huge.

### Challenges and Potential Solutions Related to the Use of AI Robots in ICUs

#### Overview

Despite significant advancements and widespread applications of AI robots across all processes within the ICU, they face a host of challenges due to the complexity and uncertainty of the real world, algorithmic limitations, and ethical and moral considerations. These challenges encompass safety, dignity, privacy, and questions of liability. In addition, the implementation of advanced technologies requires considerable investment, making cost-benefit considerations indispensable. Our objective is to develop AI robots for ICU application that uphold human values, demonstrate ethically sound behavior, and draw robust conclusions [128]. It is imperative to accurately recognize these extant issues and propose effective solutions.

The “Asilomar AI Principles,” signed at the 2017 Asilomar conference held in Asilomar, California, United States, call on global AI professionals to adhere to these principles to safeguard the interests and safety of humanity in the future [129]. The principles emphasize ethical standards and values, including privacy, security, fairness, and transparency. Although these principles only provide an ethical framework and guiding principles for AI development and do not offer specific policies, regulations, or standards, they still serve as reference guidelines for the development of the AI field.

#### Security Issues

With the continuous evolution of AI robotic technology, its capabilities and functions are constantly being enhanced. However, there is a lack of clear standards to define the meaning of safety and accuracy and evaluate its specific programs. Narrow technical approaches are insufficient to ensure the safety of AI robots, which must be considered within the broader sociotechnical context in which they operate [130]. Therefore, systems with moral and social reasoning capabilities are becoming increasingly important. In some cases, human involvement can serve as a constraint on robot design, especially in decisions involving life and death. However, in the realm of high-speed decision-making, robots require built-in moral and social reasoning capabilities [131]. In addition, the lack of sample size, heterogeneity of diseases, and complex operations lead to biases in AI algorithms, which cannot ensure the safety and effectiveness of robot treatments. These challenges should be evaluated alongside the risks already familiar to humans, allowing us to set realistic expectations and foresee significant advancements in the realm of robot safety.

In an effort to confront the emerging safety challenges within the AI domain, various countries have embarked on proactive measures aimed at addressing these potential concerns. Initiatives such as the AI Foundation Model working group established by the United Kingdom in April 2023 [132] and the series of AI white papers updated annually by the Chinese Association for Artificial Intelligence are significant steps toward understanding and addressing these issues [133]. Moreover, the global AI Safety Summit held in November 2023 marked a pivotal moment, with 28 countries coming together to sign the “Bletchley Declaration.” This declaration represents a unified commitment to scrutinize the risks associated with the frontiers of AI technology, such as natural language processing, computer vision, and reinforcement learning, with a specific focus on the development of large language models by leading companies such as OpenAI, Meta, and Google [134]. These concerted efforts underline a global recognition of the complexities and challenges posed by AI, as well as a determined move toward collaborative solutions. By focusing on risk assessment, ethical standards, and safety protocols, these initiatives highlight an international resolve to navigate the advancements of AI technology in a manner that not only benefits human society but also safeguards against potential hazards.

#### Dignity Issues

Medicine has always been a humanistic science where physicians are expected to not only adopt a scientific attitude toward patients but also resonate emotionally with them, embodying empathetic care. Patients in the ICU typically exhibit both physical and psychological fragility, necessitating humanistic care and emotional support from medical staff. This cannot be substituted by a robot due to its “mechanical”; “pre-programmed”; and, thus, “neutral” way of interacting with patients. Emotional recognition technology can be incorporated into AI robotic systems, providing corresponding emotional support by recognizing patient emotions, for instance, through voice, facial expressions, or body language. AI robots often have an unequal communication with patients, leaning more toward 1-way output from the robot. Before engaging in the discourse on equality between humans and AI robots, it is crucial to address a foundational question: should AI be classified as a tool or an agent? This distinction becomes especially pertinent in the context of conversational AI robots. When perceived merely as tools, there is a risk of undervaluing the anthropomorphic attributes and functionalities they embody. Conversely, viewing them as agents presents its own set of challenges as they inherently lack humanlike qualities such as empathy, intentionality, and the capacity to bear responsibility [135]. This debate is not new but remains central to the evolving conversation around AI’s role in society. Recent technological developments suggest that, by integrating natural language processing and voice recognition technologies, robots can become more anthropomorphic and capable of responding to patients’ language needs, alleviating feelings of loneliness and neglect in patients in the ICU. For example, the integration of the popular ChatGPT with AI robots may enhance their linguistic potential. Intelligent interactive AI robots combining ChatGPT’s linguistic skills with the computer vision and tangible abilities of robots could revolutionize the way humans interact with technology [136]. They could be better at navigating the subtleties of human interaction, boasting superior natural language generation capabilities.

On the other hand, the narrow technical AI safety field lacks ideological and demographic diversity, leading to a lack of breadth and rigor in knowledge. Moreover, practitioners in this field often come from White, male, and underrepresented groups, which is insufficient to meet the broad participation and shared human care needed for technological development, potentially leading to racial, gender, and other biases in technology application [137,138]. For instance, using Framingham Heart Study data to predict cardiovascular event risk in people of color may lead to both overestimation and underestimation of risk [139]. Similar racial biases may inadvertently be built into health care algorithms. Therefore, it is necessary to expand the space for broader participation, pursuing equality and common development from the source of technology, to avoid defects in AI robot products [140].

#### Privacy Issues

To enhance the monitoring of patients who are critically ill, robots are often equipped with surveillance apparatuses to log pertinent data and wirelessly transmit information. Such actions may infringe upon the privacy rights of patients, jeopardizing their confidentiality. Nonetheless, these features also hold merit in ensuring patient safety [141]. As AI robots proliferate across various sectors, including health care, concerns over privacy safeguards have been a recurrent topic of critique. It is imperative that AI robots adhere to privacy regulations when handling medical data, such as the General Data Protection Regulation that is prevalent in Europe [142]. Encryption techniques should be used during data collection and storage, narrowing the scope of data acquisition and ensuring anonymization. Of paramount importance is the establishment of stringent access control mechanisms, ensuring that data are accessible only to authorized personnel. Concurrently, it is vital to instate ethical guidelines and standards focused on privacy protection, which will dictate the conduct of AI robots and their data-handling procedures. All these regulations must ensure that individuals retain a voice over the collection, storage, and use of their information [143].

#### Attribution of Liability Issues

The integration of AI into robotic systems has rendered questions of accountability more intricate. Endowing robots with autonomy and decision-making capabilities stands as a primary objective of AI integration into robotics. However, given the current trajectory of technological advancements, intelligent analytics still bear systemic decision-making risks. When malfunctions occur, attributing responsibility for the robot’s actions becomes contentious given that robots lack comprehension of reprimand, sanctions, accountability, or remorse [144,145]. Consequently, the liability arising from erroneous decisions made by robots can pose significant legal conundrums [141]. Accountability in computer science encompasses a multifaceted domain, probing the responsibility attribution across its creation, dissemination, and use phases [146]. Thus, devising a legal framework pertinent to AI robots is essential, delineating the responsibilities and obligations of robot manufacturers, operators, and users spanning every facet of robot design, production, operation, and use. Regulatory bodies also play a pivotal role as rigorous supervisory protocols and technical benchmarks are needed to scrutinize and accredit AI robot designs and operations. Penalties and sanctions become indispensable components of this framework. Introducing a robotic liability insurance system might also serve as an efficacious remedy to mitigate the damages and risks incurred by robots. Manufacturers, operators, and users could opt for such liability insurance, necessitating clear demarcations of responsibility. Meanwhile, both the United States and the European Union advocate for a focus on algorithm transparency and accountability, aiming to make their decision-making processes more transparent and comprehensible. This will facilitate a better assessment of responsibility and serve as a warning against outsourcing moral responsibility to algorithms [147]. This calls for collaboration between manufacturers and developers, with the National Institute for Health and Care Excellence in the United States requiring developers to program code in an environment of technological feasibility and respect for intellectual property rights, ensuring reproducibility and error checking [148].

Addressing the issue of robotic accountability necessitates a holistic perspective, incorporating technical, legal, ethical, and societal dimensions; fostering collaborative mechanisms with multistakeholder participation to collectively bear the decision-making risks and responsibilities; and ensuring the utmost protection of the interests and safety of robot users and beneficiaries.

The United Kingdom has introduced the Code of Conduct for Data-Driven Health and Care Technology, adopting a “Regulation as a Service” model. This innovative approach ensures that regulatory checks are embedded at various stages throughout the AI development cycle, aiming to uphold high standards of safety, efficacy, and ethics in the creation and implementation of AI technologies in health care [149]. In June 2021, the US Government Accountability Office released an AI accountability framework covering 4 aspects: governance (promoting accountability by establishing processes to manage, operate, and oversee implementation), data (ensuring quality, reliability, and representativeness of data sources and processing), performance (producing results consistent with program objectives), and monitoring (ensuring reliability and relevance over time). This accountability framework sets principles and directions for future legislation and policy making and also serves as a model for the advancement of accountability systems in other countries [150].

#### Cost-Benefit Issues

Economic viability remains a pivotal facet in the societal integration of any nascent technology, with the deployment of AI robots in ICUs presenting intricate cost-benefit deliberations. Being an avant-garde technology, the initial research and development expenditures for AI robots are considerable. Once successfully developed and incorporated into ICU settings, they are further subjected to financial burdens, encompassing but not confined to the costs of system enhancements and upgrades; resource use during staff training and acclimatization; and investments necessitated by data security, personal dignity, privacy safeguards, and responsibility allocation issues. However, when observed from a utility standpoint, the application of AI robots bears significant value, especially for populous nations such as China. In the face of scarce grassroots medical resources, AI robots serve as catalysts, facilitating the dispersion of health care provision to underserved and remote locales, meeting the medical needs of a vast populace—this aligns with the principal objective underpinning this technological advancement. In addition, the integration of AI robots can effectively bridge disparities in health care accessibility between high- and low-income nations, substantially augmenting the societal benefits of this technology [151]. Therefore, despite the steep initial investments and continual operational expenses, in the broader spectrum of health care service provision and societal equity realization, AI robots undoubtedly offer pronounced advantages and value.

## Discussion

### Principal Findings

AI robots have firmly established their significance within intensive care, with their integration into the ICU regimen continually deepening. This paper delineates 5 distinct application domains of AI robotic systems, be it experimental or commercial, within the ICU, addressing both technical impediments and prospective research avenues while proposing potential remedial strategies. The trajectory for AI robots within the ICU setting is promising. Currently at the nascent phase of AI robot technological deployment, there remains an extensive scope of endeavors to be pursued and myriad challenges to be surmounted. As the propagation of pertinent technologies ensues, health care professionals should welcome such intelligent implementations with optimism, recognizing the present-day confines of AI apparatuses synergistically amalgamating human and system intellect, thereby maximizing the data analysis, promptings, and recommendation proficiencies of intelligent robots. Today’s robotic entities coalesce seamlessly with AI, where sophisticated robotic systems, characterized by safety and flexibility melded with augmented computational proficiencies, yield invaluable big data insights. Simultaneously, anthropomorphic designs provide patients in the ICU with a more comforting medical experience.

“There are plenty of areas in critical care where it would be extremely helpful to have efficacious, fair, and transparent AI systems,” notes Gary Weissman, assistant professor in pulmonary and critical care medicine at the University of Pennsylvania Perelman School of Medicine [152]. Similarly, Dr Brijesh Patel, an intensivist at the Royal Brompton Hospital in London, emphasizes that “intensive care is a specialty with special prospects for AI. After all, the ICU is a space where a large amount of data is routinely collected, making it an ideal place for deploying machine learning techniques” [14]. Dr Patel, who dedicates a considerable portion of his ward rounds to adjusting ventilator settings, points out that the continuous advancement in AI technology could automate such repetitive tasks.

However, there is a consensus among experts that AI is not poised to replace physicians entirely. Instead, it is seen as a tool to streamline certain tasks, enhancing efficiency where it is most needed. Aldo Faisal, a professor of AI and neuroscience at Imperial College London, emphasizes a balanced perspective on AI’s role within health care teams. He advocates for a realistic understanding of AI’s capabilities and limitations, suggesting that neither undue fear nor excessive reverence is helpful [153].

This paragraph presents a futuristic scenario representing the potential evolution and future blueprint of AI robotics in the ICU ward. Envision a scenario where a patient who is critically injured is admitted to the ICU and greeted by “IntelliGreet”—an intelligent reception robot that collates the patient’s fundamental data and medical history, seamlessly completing admission formalities. The therapeutic reins are taken over by “MediBot,” a state-of-the-art medical robot that executes an array of treatment procedures (ranging from drug administration and wound care to life support) predicated on the patient’s condition and physician’s directives. The patient’s nursing needs are catered to by “CareCompanion,” an omnipresent nursing robot that meticulously monitors vital signs, offering essential care services (such as cleaning, feeding, and movement) while concurrently assuming the responsibility of reporting to physicians. As the patient recuperates under their meticulous care and nears discharge, “DischargeDuty” steps in, formulating discharge plans and subsequent treatment regimens based on physician prescriptions, facilitating communication with both the patient and their kin, and overseeing the discharge processes. Finally, the domestic care robot “HomeCareHelper” persists in its caregiving, administering medication reminders, monitoring patient health, and even offering rudimentary domestic aid for solitary individuals. All AI robots in this envisioned setting are interlinked via a cloud data platform, enabling real-time sharing of patient medical data, achieving cohesive and synergized medical service delivery. Reflecting upon the advancements in AI robotic technology over recent decades, it is undeniable that this vision is poised to materialize.

### Limitations of This Review

This review acknowledges a number of limitations that could affect the interpretation and applicability of its findings. One significant concern is the potential for publication bias, a common issue in scientific literature where studies with negative results are less likely to be published. This could lead to an overrepresentation of positive findings in this review. In addition, despite efforts to mitigate bias by involving interdisciplinary experts and employing dual reviewers during the literature search and data collection phases, subjective biases could still influence the selection and interpretation of the studies.

Another challenge is the absence of a universally accepted classification system for ICU robotic systems. In response, our classification framework was developed based on expert opinions and existing literature, striving for as comprehensive and rational an approach as possible. Nevertheless, the potential for omissions exists given the rapidly evolving nature of technology and the diverse applications of robotics in critical care settings. These limitations highlight the need for ongoing research and critical evaluation of emerging technologies in health care, emphasizing the importance of transparency and methodological rigor in scientific reviews.

### Conclusions

This scoping review comprehensively covered AI robots in the ICU, detailing the most widely used or newly developed robotic devices on the market. Robots in ICU wards are becoming valuable assistants to physicians and nurses. Although ethical and safety concerns remain unresolved in this field, these challenges are inevitable in the development of new technologies, and experts and developers are focusing on addressing them. Future research should focus on developing policies and regulations to prevent or resolve these issues, making AI robots an integral part of ICUs and other hospital wards.

## Appendix

Multimedia Appendix 1 PRISMA-ScR (Preferred Reporting Items for Systematic Reviews and Meta-Analyses extension for Scoping Reviews) checklist.

Multimedia Appendix 2 Classification of intensive care unit robots.

## Abbreviations

5G: fifth-generation
AI: artificial intelligence
CPR: cardiopulmonary resuscitation
CRISPR: Clustered Regularly Interspaced Short Palindromic Repeats
ICU: intensive care unit
KINARM: Kinesiological Instrument for Normal and Altered Reaching Movements
MGIUS-R3: MGI Ultrasound System-Remote 3
NICU: neonatal intensive care unit
PRISMA: Preferred Reporting Items for Systematic Reviews and Meta-Analyses
PRISMA-ScR: Preferred Reporting Items for Systematic Reviews and Meta-Analyses extension for Scoping Reviews
RP-7: Remote Presence-7
RP-VITA: Remote Presence Virtual + Independent Telemedicine Assistant
SCI: spinal cord injury
VR: virtual reality
