# Supplementary material for: Advances in the Application of AI Robots in Critical Care: Scoping Review
Source: J Med Internet Res. 2024 May 27;26:e54095. doi: 10.2196/54095 (PMC11165292; doi:10.2196/54095)
Supplement: Multimedia Appendix 2 [file jmir_v26i1e54095_app2.docx]

**Table S1. Classification of ICU robots**

| Classification | Definition | Equipment | Structure | Function | Time | Country | Certification | References |
| --- | --- | --- | --- | --- | --- | --- | --- | --- |
| Therapeutic auxiliary robots | Therapeutic auxiliary robots are a type of robotic devices that provide assistance and support in medical settings. | Ventilator-Mounted Cartesian robot | • Ventilator touch screen with camera vision control; • Maquet Servo-U ventilator; • a two-axis gantry; • a mechanized end-effector finger; • a pair of 45 Ncm NEMA 17 stepper motors; • a 15 TFT LCD capacitive touchscreen. | The teleoperated ventilator controller system consists of a custom robotic patient side device and a touch-based master console. Computer vision tasks that enable an intuitive user interface and accurate robot control are executed on the master. | 2021 | USA | In Testing Phase | 6, 18-21 |
|  |  | McSleepy | • Automatic control system; • Drug delivery system; • Sensor for measuring muscle movement; • Monitoring module system; • The graphical user interface and "brain" of the system. | McSleepy is an anaesthesia robot capable of performing automatic closed-loop anaesthesia-delivery to control the induction, maintenance and arousal of general anaesthesia. | 2013 | Canada | Applied clinically | 21-28 |
|  |  | Cardiopulmonary Resuscitation Robot (CPR) | • Robot manipulator for performing cardiopulmonary resuscitation; • Integrated user interface; • Monitoring equipment. | An automated robot CPR system can perform CPR automatically, analyze the patient’s condition, and relay the information to the CPR system. | 2016 | Republic of Korea | Applied clinically | 29-32 |
| Nursing auxiliary robots | Nursing care robots are designed to assist bedridden patients with simple services. | VenousPro™ | • 7-degree-of-freedom venipuncture system (a 3-degree-of-freedom (DOF) gantry system attached to a 4DOF injection arm); • Near infrared and ultrasonic imaging systems; • Image analysis software; • Three-dimensional vein reconstruction software. | VenousPro™ is an automated robotic venipuncture device designed to identify suitable vessels for cannulation and robotically guide an attached needle towards the lumen center, which allows blood drawn from peripheral forearm veins safely. | 2014 | USA | Commercialized | 33-38 |
|  |  | sputum suction robot | • 6DOF robotic arm; • Manipulator and clamping device for imitating metacarpophalangeal joints. | The sputum suction robot is stable in movement and can smoothly complete the clamping, feeding, back-off protection, and rotating tube removal of the sputum suction tube to achieve effective sputum suction. | 2019 | China | In Testing Phase | 39 |
|  |  | Calmer | • A robot equipped with a surface mattress resembling skin. | Calmer was developed to manage acute pain effectively for preterm infants in the neonatal ICU by simulating key pain-reducing components of human touch-based treatment. | 2019 | Canadian | In Testing Phase  (PCT Utility Patent # CA2015/051002) | 40,41 |
|  |  | PARO | •PARO resembles a petite, white seal adorned with pronounced, dark eyes and a plush exterior. | PARO is receptive to tactile stimuli, auditory inputs, and light variations. It exhibits capabilities such as tail oscillation, auditory emission, and cranial movement to facilitate interaction with users. The primary intent behind its conceptualization is to serve as a conduit for emotional solace and therapeutic alleviation, with an emphasis on geriatric demographics and medical patients. | 2003 | Japan | Commercialized | 42-45 |
| Rehabilitation auxiliary robots | Rehabilitation robots are aimed at facilitating the recovery of impaired sensory, motor, and cognitive skills. | Lokomat | • Treadmill into a dynamic support system; • An all-robot orthosis in collaboration with; • Equipment control system. | Lokomat is a robotic gait training system. It is used for gait training for patients with abnormal gait caused by brain injury, spinal injury, neurological injury, muscle injury, and orthopedic diseases and improves the mobility of patients with neurological diseases. | 2005 | Hocoma, Switzerland | Approved by the FDA (3003781275) | 46-58 |
|  |  | ArmeoSpring | • An adjustable suspension system for the upper limb; • A system for detecting grip pressure; • Controls the functionality settings interface. | ArmeoSpring is an arm fixed frame rehabilitation exoskeleton. The device can be used as an evaluation tool for the capabilities and range of the user’s arm. Sensors can also be used to provide interactive training and integration with virtual reality. | 2007 | Hocoma, Switzerland | Commercialized | 59-63 |
|  |  | KINARM | • Kinarm Exoskeleton robots; • 2D virtual/augmented reality display; • Dexterit-E™ experimental control software and hardware; • Optional Kinarm Gaze-Tracker™; • System-integrated chair with wheelchair-style seating. | KINARM is a robotics research tool designed to make quantitative neurological assessments of sensorimotor, proprioception, and cognitive brain function. | 1999 | BKIN Technologies, Canada | Commercialized （U.S. Patent No. 6,155,993; 8,347,710 & 8,800,366; Canadian Patent No. 2,267,821; EP No. 2,150,175） | 64-68 |
| Telepresence robots | Telepresence robots use Al technology and man-machine interaction to provide adjuvant diagnosis and treatment of the doctor's professional technology. | RP-7 | • Wireless mobile devices that are approximately 5.5 feet tall overall; • A panoramic Virtually There visualization system; • A convenient mobile overall drive system; • Monitor the proximity of hazardous Sense Array System 360; • At the top is a flat screen with a control interface; • Oral sound control loudspeaker convenient for real-time oral communication. | RP-7 is a real-time audio and visual robotic telepresence system that provides communication between patients, hospital staff, and a remote physician. | 2007 | USA | Approved by the FDA (K073710) | 69-74 |
|  |  | Stevie | • Consists of a "human-like" head (social interface and status communication indicators), trunk, and arms; • Two onboard computers (Intel NUC); • The bottom is provided with wheels supporting omnidirectional; • Suspension of combined leaf spring and air spring; • Private earpiece and stethoscope port; • Externally covered with a layer of soft textile fabric; • a RGBD depth sensor, 360 camera, and microphone array; • A two-dimensional lidar (Hokoyu URG) sensor. | Stevie has a stethoscope port and a high-definition pan-tilt-zoom camera, which can relay information during an examination of a patient and help doctors identify illnesses and diseases in the ICU. | 2021 | Sweden | Applied in Steve Biko academic hospital | 75, 76 |
|  |  | MGIUS-R3 | • A physician-side subsystem (a virtual sensor, a copy control panel of the ultrasound instrument, an ultrasound image monitor, a hand-held controller with six degrees of control freedom); • A patient-side subsystem (a 64-channel portable ultrasound system, a 6-of-freedom robotic arm, a forcesensor equivalent with convenx and linear array transporters, tele-echography system); • A high-definition audio-video communication system. | MGIUS-R3 is a robot-assisted teleultrasound diagnostic system. Doctors could manipulate the robotic arm and adjust parameters to perform teleultrasound examinations for real-time transmission of audio, video, and ultrasound images by 5G network technology. | 2021 | China | In Testing Phase | 77-80 |
|  |  | 5G+ medical robot +VR visiting system | • 360-degree 8K panoramic camera; • 8K VR eyewear; • High definition remote video interactive system; • 5G panoramic VR real-time display system. | The system makes use of 5G networks, medical robots, 360-degree 8K panoramic cameras, 8K resolution VR glasses and other technical means to enable remote visits to patients. | 2021 | China | Applied in West China Hospital of Sichuan University | 81 |
| logistics and disinfection robots | Transport robots are used to transport medical supplies, drugs, patients, etc. Disinfection robots are used for ward other space sterilization. | Helpmate | • 28 ultrasonic ranging sensors with different orientations; • Structured light sensor system; • Autonomous navigation sensors; • Man-machine interface (screen and keyboard and a voice output system). | HelpMate is a robotic materials transport system designed for hospitals. Helpmate mainly navigates based on pre-stored maps and has a certain obstacle avoidance ability. | 1989 | USA | Approved by the FDA (K933633) | 82 |
|  |  | APS II Robot-Rx | • A cart fix to that drug delivery; • Automatic robot drug delivery system. | APS II Robot-Rx is a pharmacy delivery robot in intensive care units. It drives the 24-hour cart fill, which is supplemented by unit-based cabinets in patient care areas, clinics, and procedure areas. | 2003 | USA | Applied clinically | 83, 84 |
|  |  | c-PAM | • An upper conveying plate and a lower conveying plate with a conveying belt; • Drive motor and controller. | c-PAM is a robot for patient transfer mainly through a conveyor belt to complete the patient transfer between the bed and the stretcher. | 2007 | Japan | Applied clinically | 85 |
|  |  | Xenex robots | • Pulsed Xenon UV disinfection system | The Xenex robots are disinfection robots by using pulsed xenon to create intense bursts of broad-spectrum UV light. | 2015 | USA | Commercialized | 86 |
|  |  | EPS logistics disinfection robot | • ultraviolet spectra lamps; • 3D camera; • Laser sensor navigation; • Intelligent sensing system. | EPS logistics disinfection robot has transport and disinfection functions. It undertaking the drug distribution work of nurses from the station to the ICU ward of patients. At the same time, it can customize the disinfection time and disinfection route for high-frequency areas of doctor-patient activity and can carry out automatic UV disinfection. | 2018 | China | Commercialized | 87 |
|  |  | HPV disinfecting robot | • 5G temperature patrol robot system: • Infrared temperature measurement screening and prevention and control command system. | The HPV disinfecting robot combines a hydrogen peroxide device with a robot. The disinfecting system inside the robot generates disinfecting gas and can realize autonomous navigation and autonomous movement in an unmanned environment. | 2020 | China | Used for the prevention and control of the new crown virus pneumonia epidemic | 88, 89 |
|  |  | a novel robot system | •The system comprises an automatic guided vehicle (AGV), an air sample collector, and a pathogen detection system | By autonomously patrolling and collecting air samples, the robot utilizes biosensor technology to perform real-time detection of airborne pathogens | 2022 | China | Design phase | 90 |
